# A Three-Dimensional Elastic-Plastic Contact Analysis of Vickers Indenter on a Deep Drawing Quality Steel Sheet

**DOI:** 10.3390/ma12132153

**Published:** 2019-07-04

**Authors:** Tomasz Trzepiecinski, Hirpa G. Lemu

**Affiliations:** 1 Department of Materials Forming and Processing, Rzeszow University of Technology, al. Powst. Warszawy 8, 35-959 Rzeszów, Poland; 2 Faculty of Science and Technology, University of Stavanger; N-4036 Stavanger, Norway

**Keywords:** numerical modeling, finite element method, hardness, material properties, surface properties, Vickers hardness

## Abstract

Three-dimensional finite element-based numerical analysis of Vickers indenter hardness test was conducted to investigate the effect of frictional conditions and material anisotropy on indentation results of deep drawing quality steel sheets. The strain hardening properties and Lankford’s coefficient were determined through the uniaxial tensile tests. The numerical computations were carried out using ABAQUS nonlinear finite element (FE) analysis software. Numerical simulations taken into account anisotropy of material described by Hill (1948) yield a criterion. The stress and strain distributions and loading–unloading characteristics were considered to study the response of the material. It was found that the hardness values seemed to be influenced by the value of the friction coefficient due to the pile-up phenomenon observed. The increasing of the friction coefficient led to a decrease of the pile-up value. Moreover, the width of the pile-ups differed from each other in the two perpendicular directions of measurement. Frictional conditions did not significantly affect the maximum force and the character of load–displacement curves. Frictional regime between the indenter and workpiece caused that the region of maximum residual stresses to be located in the subsurface.

## 1. Introduction

The wide interest in hardness, as a significant property from the technological point of view, results in the development of many different measurement methods. It should be emphasized that the hardness together with the yield stress and ultimate tensile strength allows us to characterize the mechanical properties of the specific alloy or to control the correctness of the technological processes carried out. The most popular are the penetration methods, consisting in penetrating the indenter (of different shape) into the material, until permanent deformation is obtained in the form of an impression whose size together with the value of forces (which caused permanent deformations) are the basis for determining the hardness in units that are characteristic for a given measurement method. Hardness measurement methods are comparative methods and are indirect. The variety of hardness measurement methods results in the fact that the obtained hardness measurements between different methods obtained in various ways are in most cases incomparable [1,2].

In general, direct comparison of hardness values obtained with different methods (conversions) is not possible, especially in the case of hardness units defined differently. Moreover, for all penetration methods, the rule of similarity is governed, which allows us to compare the results of hardness measurements only if the similarity of impressions is preserved (the so-called Kick’s similarity law).

Many indentation tests have been developed to study the mechanical properties of materials. The most used are standardized methods of Vickers, Rockwell and Brinell. The simplicity and speed of evaluating the plastic and elastic properties of metallic materials makes the indentation tests one of the powerful tools for characterization of bulk and thin film materials. Some scholars have studied the relationship between hardness and other mechanical properties, such as elastic modulus [3,4], yield strength and ultimate tensile strength [5]. A critical review of indentation hardness measurements at different scales has been presented by Broitman [6]. In recent years, many authors examined the effect of different finite element (FE)-model parameters, indented geometry and material tested elastic-plastic description on the load–indentation curve course and hardness value. Most of them use two-dimensional axisymmetric analyses with conical and spherical indenters [7,8]. The Vickers indenter is not axisymmetric. However, some authors studied the Vickers indentation process as two-dimensional axisymmetric model [9]. Dao et al. [10] conducted a review analysis on the data derived from indentation by FE-based simulations using 2D axial symmetric model instead of the real configuration.

In the last decades, three-dimensional numerical simulations of the Vickers test were used to study the effect of the indentation test on the mechanical property results. Antunes et al. [11] developed finite element simulation software, HAFILM, to simulate the ultramicrohardness tests. Different mesh refinements were tested because of the dependence between the values of the mechanical properties and the size of the finite element mesh. Another parameter studied in this work is the value of the friction coefficient between the indenter and the sample using numerical simulation. In order to obtain numerical results close to reality, a common geometry and size of the imperfection of the tip of Vickers indenter was taken into account for the numerical description of the indenter.

Recently, the crystal plasticity finite element method (CPFEM) was developed to investigate the anisotropic mechanical behaviors of single-crystalline undergoing nano-indentation. Liu et al. [12] developed a CPFEM model to investigate the effect of the coefficient of friction on the evolution of crystallographic texture and mechanical behavior of the initially oriented aluminum single crystal during nano-indentation. The piling-up curve was captured on the deformed surface and it decreased when the coefficient of friction increased. The analysis also revealed that these lattice rotation angles in the chosen deformed zone were significantly affected by the friction coefficient during nano-indentation. Yeo et al. [13] developed a combined experimental and modeling approach to study the indentation damage test on the thin-film stacked structures. The modeling of the thin-film stacked structures under indentation loading and unloading processes has been conducted to analyze the stress field and explain their indentation damage mechanisms. The results provides an understanding of the indentation damage mechanisms of the thin-film stacked structures, where cracking normally occurs at the brittle Si substrate or/and at the intermediate layer underneath the top metal layer.

Antunes et al. [14] performed numerical simulations to estimate the influence of the size of the Vickers tip imperfection on the hardness and Young’s modulus results. Among others, they found that the load-unload curves are independent of the friction coefficient. Sakharova et al. [15] performed the three-dimensional simulations of Berkovich, Vickers and conical indenter to evaluate the effect of indenter geometry on the load–displacement curves and hardness value of bulk and composite materials. One of the main conclusions is that the equivalent plastic strain distributions depend on indenter geometry. Libório et al. [16] used the FE method to simulate the Vickers indentation test to numerically assess the penetration depth as a function of the hardness. They also proposed a simple experimental–numerical methodology to determine the thickness of the physical vapor deposited films by comparing the numerical results with experimental Vickers hardness testing. Simion et al. [17] used a 3D numerical simulation of micro-indentation test using a Vickers indenter was performed to determine the geometrical parameters of imprint after elastic recovery and to estimate the Vickers microhardness. Madeiros and Dias [18] analyzed numerically the mechanical behavior of a specimen of tungsten carbide cobalt during a Vickers indentation test, based on FE computations. Giannakopoulos et al. [19] and Larsson et al. [20] analyzed both Vickers and Berkovich indenters. They found that although the geometry functions of Berkovich indenter and Vickers indenter can be designed to be the same as that of the conical indenter, the true projected contact areas at the same indentation depth may still be different considering the effect of pileup. The effect of pileup on the accuracy of sharp indentation testing has been studied by Tang et al. [21] by means of numerical simulation. However, the results obtained might by valid for a group of specific materials whose strain hardening is ignored and the effect of the Poisson’s ratio is not considered.

However, the many experimental and numerical studies have been devoted to the analysis of contact phenomena in indentation tests, the sensitivity of the numerical results on contact conditions, material anisotropy and strain hardening phenomenon still needs further investigation. To our knowledge there are no investigations of indentation concerning the simultaneously both effect of the friction conditions, real strain hardening phenomena in the regime of small deformations and material anisotropy. In this paper the effect of frictional conditions and material properties on results of three-dimensional numerical simulations of Vickers indentation of deep drawing quality steel sheets is studied.

## 2. Theoretical Background

In the Vickers hardness measurement method conducted according to the EN ISO 6507-1:2018 standard [22] a diamond indenter, in the form of a right pyramid with a square base and an angle of α = 136 ± 0.5 degrees between opposite faces is subjected to a load with specific value. This hardness measurement method is developed by Smith and Sandland in 1925. It received the Vickers designation due to the fact that they were working in a company called Vickers Ltd. Depending on the indentation load, the four ranges of the Vickers scale are distinguished: Nanohardness—load value below 0.1 g, microhardness—load value to 200 g, hardness at small load—load value between 200 and 1000 g and macrohardness—load value in the range of 1000 to 1.2 × 10^5^ g. The full load is normally applied for 10 to 15 seconds. The hardness of Vickers scale is calculated from the formulae:

(1) $$HV=0.102\frac{P}{A},$$

where *P* (N) is the applied load, *A* (mm^2^) is area of the sloping surface of the indentation calculated based on the average *d* (mm) of two diagonals *d*_1_ (mm) and *d*_2_ (mm) of the indentation left in the surface of the material after removal of the load. Thus, the area is calculated from:

(2) $$A=\frac{1}{2}\frac{d^{2}}{sin\frac{136^{o}}{2}}.$$

After combining (1) and (2) the expression in (1) takes the form:

(3) $$HV=\frac{0.204Psin68^{o}}{d^{2}}.$$

The hardness test also allows us to evaluate the reduced Young’s modulus of the material tested according to the contact area and the measured unloading compliance:

(4) $$E=\frac{1}{\beta }\frac{\sqrt{\pi }}{2C}\frac{1}{\sqrt{A}},$$

where A is the contact area (mm^2^), *C* (mm·N^−1^) is the compliance, *β* is a correction factor which depends on the indenter’s geometry, where *β* varies in the range 1.01–1.07 for Vickers indenter [9,11]. 

The reduced Young’s modulus is a function of the Young’s moduli and Poisson ration of the specimen and indenter material:

(5) $$E=\frac{E_{s}E_{i}}{E_{i}\left(1-\nu_{s}^{2}\right)+E_{s}\left(1-\nu_{i}^{2}\right)},$$

where *E_i_* (MPa) and *E_s_* (MPa) are Young’s moduli of indenter and specimen material, respectively; *ν_i_* and *ν_s_* are Poisson’s ratios of indenter and specimen material, respectively.

The compliance *C* may be obtained by differentiating the equation [23]:

(6) $$P=K{\left(h-h_{f}\right)}^{m},$$

with respect to the indentation depth *h* (mm), at the point of maximum load, and this differentiated expression is given as:

(7) $$\frac{1}{C}=\frac{dP}{dh}=mK{\left(h_{max}-h_{f}\right)}^{m-1},$$

where *K* and *m* are constants obtained in the fit to the unload curve, *P* (N) is the load, *h* (mm) and *h_f_*(mm) are depths of indentations at the current value of load and after unload, respectively [14].

According to Oliver and Pharr [24] the projected contact area *A* (mm^2^) is a function of penetration depth *h* (mm) and contact depth *h_c_* (mm):

(8) $$A=f\left(h\right)=f\left(h_{c}\right).$$

The projected contact area A (mm^2^) may be greatly underestimated if the pileup effect exists. If the pileup effect is negligible, *h_c_* (mm) can be determine by

(9) $$h_{c}=h-\frac{\varepsilon P_{max}}{S},$$

or

(10) $$h_{c}=h_{max}-\varepsilon CP_{max},$$

where *h_max_* (mm) is the indentation depth at the maximum load *P_max_* (N), *ε* is a constant that depends on indenter geometry and *S* (N·mm^−1^) is the elastic stiffness of the contact. 

The value of the geometrical parameter *ε* depends on the geometry of indenter and varies between 0.72 (conical indenter) and 1 (flat indenter). Oliver and Pharr [24] proposed an analytical method of *ε*-value evaluation based on the unload curve and effective indenter shape:

(11) $$\varepsilon =1-\left(m-1\right)\frac{G\left(\frac{m}{m-1}\right)}{\sqrt{\pi }G\left(\frac{1}{2m-2}\right)},$$

where *G* is a gamma function and *m* is the exponent in Equation (6). 

Another characteristic of load-unload curves is determination of uniaxial mechanical properties, such as yield stress and the strain hardening exponent *n* [9,25]. Johnson [26] found that hardness of ductile elastic-plastic materials is expressed by:

(12) $$\phi =\frac{E}{\sigma_{r}}tan\alpha ,$$

where *E* (MPa) is Young’s modulus of the material, α (rad) is the angle of inclination of the face of the indenter to the surface of specimen and *σ_r_* (MPa) is the representative stress associated to the representative plastic strain *ε_r_* [26]:

(13) $$\varepsilon_{r}=\frac{tan\alpha }{5}.$$

Tabor [27] found that for ductile materials Vickers hardness *HV* is proportional to the uniaxial stress, at the representative plastic strain *ε_r_* = 0.08:

(14) $$HV=3.3\sigma_{r}.$$

Casals and Alcalá [25] developed dimensionless functions related to the mechanical properties determined through stress–strain curves and load–unloading curves. Then, the load *P* (N) is proportional to the square of the indentation depth *h* (mm) and expressed as:

(15) $$P=kh^{2},$$

where *k* is the proportionality coefficient (constant).

The relation in Equation (15) is called Kick’s law. The ratio between constant *k* and representative stress *σ_r_* is independent of strain hardening exponent *n*.

**Figure 1:**
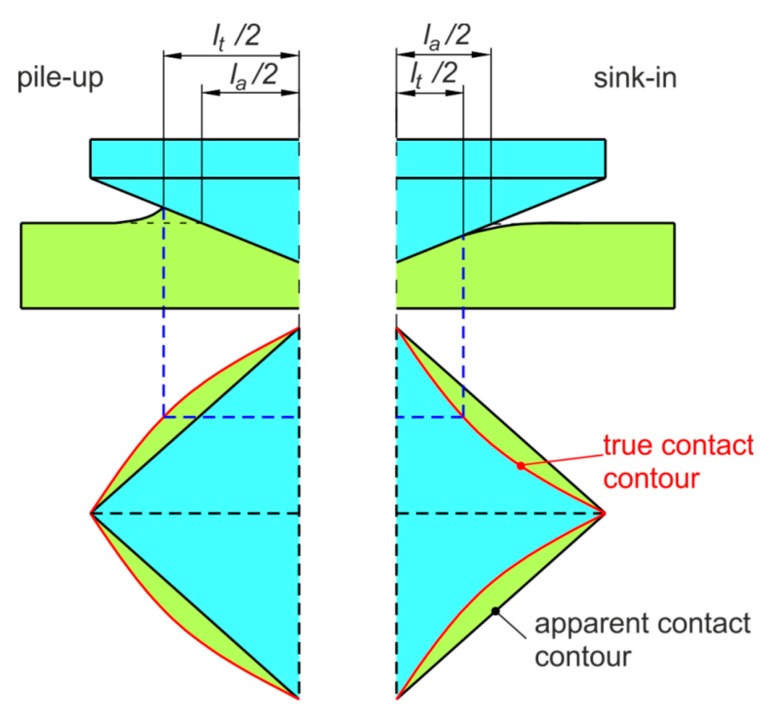
Change of the contact contour of the indent due to pile-up and sink-in: *l_a_*—apparent contact length (mm), *l_t_*—true contact length (mm).

**Figure 2:**
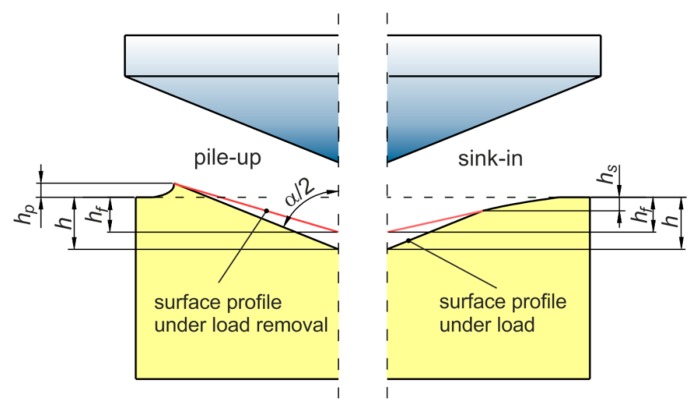
Cross section of the indentation profile, dimensions in (mm).

In the indentation process, the true contact area depends on the elastic-plastic properties of the workpiece material, type of material and grain size. Two situations may occur in normal indentation. The real area of contact depends on the values of either pile-up or sink-in behaviors (Figure 1). The pile-up effect causes the bulging of material between the indenter corners. In contrast the sink-in effect causes the pushing of material in the direction of indenter load. The pile-up height *h_p_* and sink-in depth *h_s_* (Figure 2) can vary based on the materials of the substrate and film. When the substrate is more compliant than the film, sink-in occurs [28]. The effects of the pile-up and sink-in behavior on the hardness and elastic modulus were studied by many authors [29,30]. The effect of pile-up can be correlated with the orientation of material (anisotropy).

## 3. Material

The numerical simulations of indentation tests were carried out for 2-mm-thick deep drawing quality DC04 steel sheets. The required chemical composition of sheet material according to the EN 10130:2009 [31] standard is listed in Table 1. The mechanical properties of the sheet metal (Table 2) have been determined through uniaxial tensile tests according to EN ISO 6892-1:2016 [32] standard. Specimens for tensile tests were cut along three directions with respect to the rolling direction of the sheet metal (0°, 45° and 90°). The following parameters have been determined: Yield stress *R_p_*_0.2_, uniaxial tensile stress *R_m_*, strength coefficient *K*, strain hardening exponent *n* and Lankford’s coefficient *r*. Three specimens were tested for each cutting direction and average value of basic mechanical parameters were determined.

The anisotropy of plastic behavior of sheet metals is characterized by the Lankford’s coefficient *r*, which is determined using the formula:

(16) $$r=\frac{\ln\frac{w}{w_{0}}}{\ln\frac{l_{0}\cdot w_{0}}{l\cdot w}},$$

where: *w*_0_ (mm) and *w* (mm) are the initial and final widths, while *l*_0_ (mm) and *l* (mm) are the initial and final gage lengths, respectively.

If the value of the r-coefficient is greater than 1, the width strains are dominant, which is a characteristic of isotropic materials. On the other hand, a value of r < 1 indicates that the thickness strains will dominate.

With the increase of deformation under cold forming conditions, the mechanical properties of the deformed metal change, significantly affecting the course of plastic forming operations. It is caused by material strain strengthening caused by plastic deformation. In order to take this phenomenon into account in numerical computations, the values of the strength coefficient *K* and the value of the dimensionless strain hardening exponent *n* were determined by approximation of the experimental stress–strain curve using the Hollomon power law relationship:

(17) $$\sigma_{p}=K\cdot \varphi_{i}^{n},$$

where *σ_p_* (MPa) is the yield stress and *φ_i_* is equivalent plastic strain.

**Figure 3:**
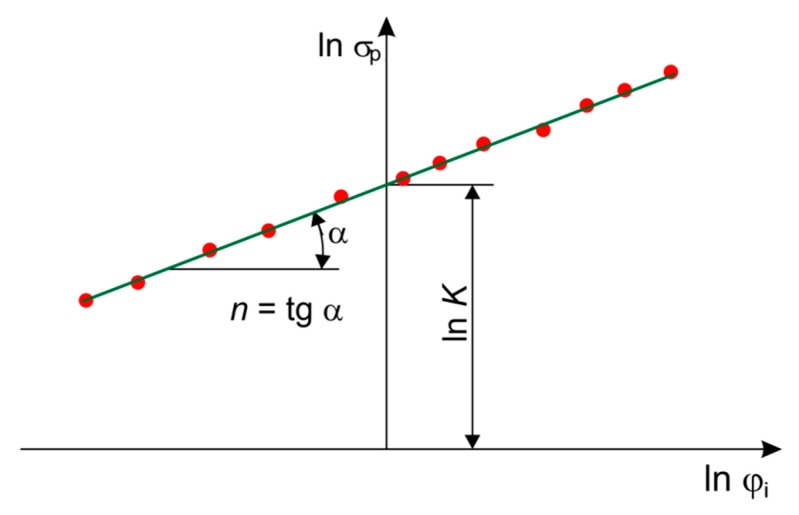
Graphical method of determination of strain hardening parameters.

In order to determine the parameters *K* (MPa) and n, a graphical method was employed. This method consists in approximation of the stress–strain data in the logarithmic coordinate system $\ln\sigma_{p}$ vs. $\ln\varphi_{i}$ (Figure 3). In the logarithmic coordinates, the strain hardening curve $\ln\sigma_{p}=\ln\sigma_{p}\left(\ln\varphi_{i}\right)$ is a straight line and takes a form:

(18) $$\ln\sigma_{p}=\ln K+n\cdot \ln\varphi_{i},$$

The directional coefficient of the straight line passing through the points plotted in logarithmic coordinate system is *n*, whereas the point of intersection of the line with the abscissa defines the value of *K* (MPa).

For each of the tensile tests, the points of the strengthening curve were approximated by a straight line whose parameters allowed us to determine the coefficients *K* and *n*. The fitting quality of the approximation line to the experimental data is assessed by the value of the determination coefficient *R*^2^:

(19) $$R^{2}=\frac{\sum_{i=1}^{n}\left({\hat{y}}_{i}-\bar{y}\right)}{\sum_{i=1}^{n}\left(y_{i}-\bar{y}\right)},$$

where: *y_i_* is the actual value of the variable at the moment *i*, $\bar{y}$ is the arithmetic mean of the explained variable and ${\hat{y}}_{i}$ is the value of the explained variable determined on the basis of the model.

**Figure 4:**
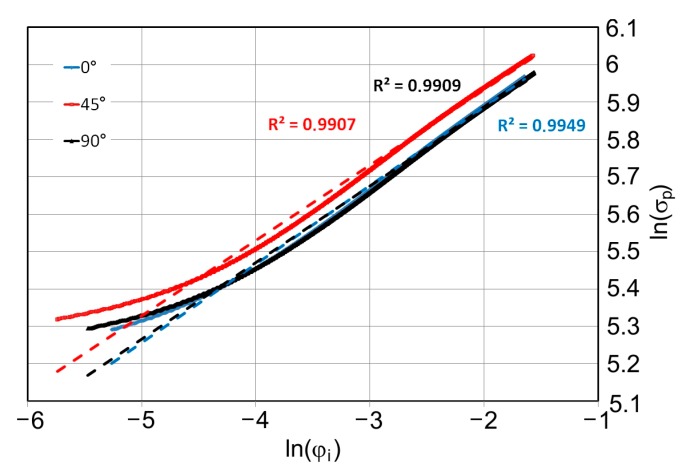
Exemplary strain hardening curves for the specimens cut at 0°, 45° and 90°.

The values of the determination coefficient *R*^2^ in the case of approximation of all experimental data was above 0.985. Examples of graphs of the strain hardening functions in a logarithmic coordinate system are shown in Figure 4.

## 4. Numerical Modeling

### 4.1. Geometry and Boundary Conditions

**Figure 5:**
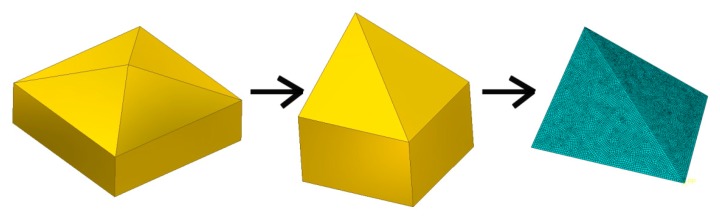
The geometry of the indenter.

**Figure 6:**
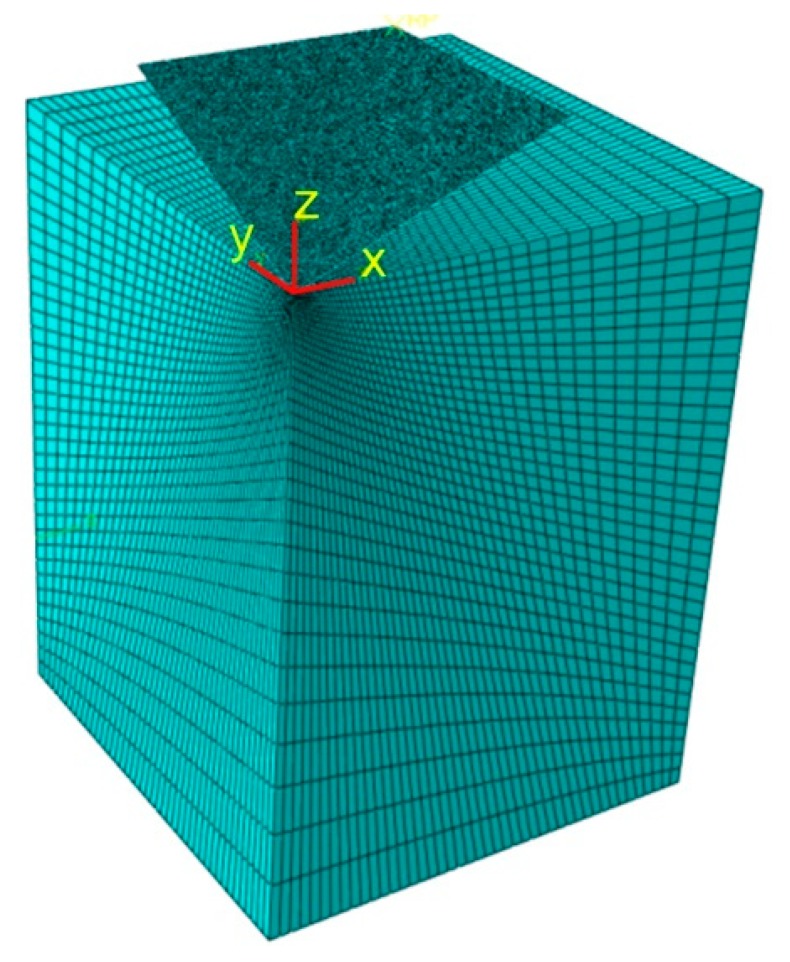
Finite element mesh used in the numerical simulation.

A 3D finite element model of the indentation tests was built using the commercial FE-package ABAQUS/Standard. Vickers indenter (Figure 5) was modeled as a 3D rigid body. Ma et al. [33] demonstrated that tip imperfection of the indenter does not have a major influence on the hardness, so, the Vickers indenter geometry in numerical modeling is assumed as ideal, without rounding. The geometry of the indenter corresponded to a surface meshed by rigid elements. Furthermore, the indenter had two planes of symmetry. In this context, one quarter of the indentation process was considered (Figure 6). The height of the model corresponded to the real sheet thickness (*t* = 2 mm). Due to geometrical symmetry in the x- and y-plane, the displacements of the appropriate nodes were fixed (Figure 6). The loading force was measured at four indentation depths: 0.025 mm, 0.05 mm, 0.075 mm and 0.1 mm.

The key to limiting the size of a computational task, while maintaining the accuracy of calculations, is the appropriate choice of the element size. Large elements will cause that the obtained results to differ significantly from reality. In contrast, too small elements may cause a considerable extension of calculations without significant increase in the computational accuracy. To establish the element size, the mesh sensitivity analysis was conducted at four mesh densities with the number of elements in the workpiece model of 46202, 79507, 124384 and 238140. In all cases, the denser mesh is assumed in the area of contact of indenter surface with the workpiece (Figure 6). The “seed edges” option in ABAQUS with specific bias ratio ψ = 33 was used to have concentration of element edges at the corner of the workpiece model.

As the parameter that is the basis for the selection of the finite element mesh size, the maximum force of indentation of the indenter at the depth of 0.05 mm under friction conditions (μ = 0.1) was assumed. Increasing the number of elements from 46202 to 79507 caused a decrease in the force to about 0.30% (Table 3). Further increase in the number of elements to 124384 and 238140 reduced the maximum load to about 0.52% and 0.526%, respectively, in relation to the model that consisted of 46202 elements, so, it was assumed that the model consisting of 46202 elements is able to accurately predict the load in reasonable time without significant loss in the prediction accuracy.

In final the configuration, the indenter surface has been meshed by 8375 elements using a 4-node 3-D bilinear rigid quadrilateral elements. The workpiece was modeled with an 8-node linear brick, reduced integration elements. These brick elements have the capability of representing large deformations and material and geometrical non-linearity.

### 4.2. Contact Conditions

The numerical model took frictional forces, which resisted the relative sliding of the surfaces of the indenter and the workpiece. The coulomb friction (CF) model is a common approach used to describe the interaction of contacting surfaces. In Coulomb friction, two contacting surfaces can carry shear stresses up to a certain magnitude across their interface before they start sliding relative to one another, and the maximum allowable frictional stress across an interface is to relate to the contact pressure between the contacting bodies. 

In general, CF model depends on the equivalent slip rate ${\dot{\gamma }}_{eq}$, contact pressure *p* and average temperature *T* at the contact point, and it defines the critical shear stress at which sliding of surfaces starts as a fraction of the contact pressure *p* between surfaces [34]:

(20) $$\tau_{crit}=\mu \cdot p,$$

where μ is a coefficient of friction.

**Figure 7:**
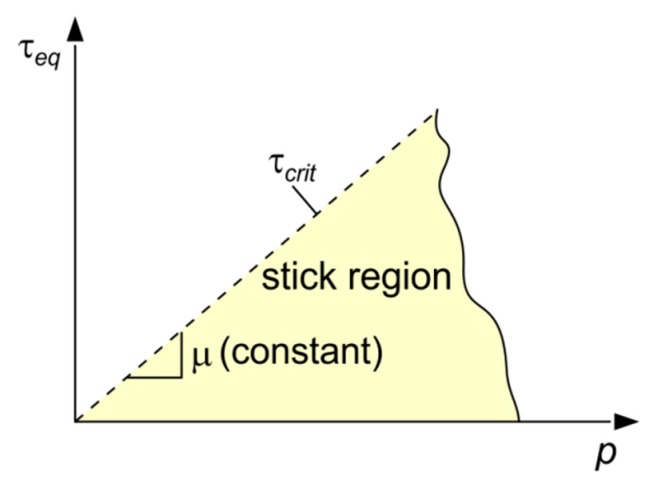
Coulomb friction model representation.

In a 3D space, the slip/stick region is represented by a surface at the contact pressure *p*, which is equivalent to the shear stress *τ_eq_* space. Figure 7 shows the two-dimensional representation of the slip region. Four coefficients of friction were studied in the numerical model of Vickers indentation: 0.0, 0.1, 0.2 and 0.3. These values correspond to the typical values of friction coefficient on DC04 steel under dry and lubricated conditions.

### 4.3. Material

The plastic behavior of the material is described by the general yield condition:

(21) $$f=\bar{\sigma }-Y,$$

where $\bar{\sigma }$ is the equivalent stress defined by the Hill (1948) [35] yield criterion developed for anisotropic metals, especially steel sheets [36,37]; *Y* is the flow stress in tension, which depends on the strain hardening exponent described by Hollomon (Equation (17)). Although the values of the determination coefficients R^2^ for the approximated strengthening curves were greater than 0.99 (Figure 2), large deviations Δ of the approximated line from the real ln(*σ_p_*)(ln (*φ_i_*)) values were observed for small strain values. Many authors do not pay attention to it. During hardness measurement, small plastic deformations usually occur, therefore it was decided in the numerical model to take into account real true stress–true strain curve.

The Hill 1948 formulation is an extension of the isotropic von Mises function, and can be expressed in terms of rectangular Cartesian stress components as:

(22) $$\bar{\sigma }=\sqrt{{\left(F(\sigma_{22}-\sigma_{33}\right)}^{2}+G{\left(\sigma_{33}-\sigma_{11}\right)}^{2}+H{\left(\sigma_{11}-\sigma_{22}\right)}^{2}+2L\sigma_{23}^{2}+2M\sigma_{31}^{2}+2N\sigma_{12}^{2}},$$

where $\bar{\sigma }$ is the equivalent stress, and indices 1, 2, 3 represent the rolling, transverse and normal directions to the sheet surface. Constants F, G, H, L, M and N define the anisotropy state of the material and are equal to:

(23) $$\begin{array}{c} F=\frac{1}{2}\left(\frac{1}{R_{22}^{2}}+\frac{1}{R_{33}^{2}}-\frac{1}{R_{11}^{2}}\right),\;G=\frac{1}{2}\left(\frac{1}{R_{11}^{2}}+\frac{1}{R_{33}^{2}}-\frac{1}{R_{22}^{2}}\right),\;H=\frac{1}{2}\left(\frac{1}{R_{11}^{2}}+\frac{1}{R_{22}^{2}}-\frac{1}{R_{33}^{2}}\right),\\ L=\frac{3}{2R_{23}^{2}},\;M=\frac{3}{2R_{13}^{2}},\;N=\frac{3}{2R_{12}^{2}}. \end{array}$$

Parameters R_11_, R_22_, R_33_, R_12_, R_13_ and R_23_ are defined from user input consisting of ratios of yield stress in different directions with respect to a reference stress according to Equation (24).

(24) $$R_{11}=\frac{\sigma_{11}}{\sigma_{0}},\;R_{22}=\frac{\sigma_{22}}{\sigma_{0}},\;R_{33}=\frac{\sigma_{33}}{\sigma_{0}},\;R_{12}=\frac{\sigma_{12}}{\tau_{0}},\;R_{13}=\frac{\sigma_{13}}{\tau_{0}},\;R_{23}=\frac{\sigma_{23}}{\tau_{0}}.$$

The elastic properties behavior of sheet material were specified using the following properties: Young’s modulus *E* = 2.1 GPa, Poisson’s ratio *ν* = 0.3 and mass density *ρ* = 7860 kg·m^−3^.

Computations were performed using the implicit finite element code. This numerical approach causes that the internal forces are balanced with the external forces through an iterative procedure, which gives the deformed state after a time increment [38]. The advantage of such an algorithm is that the time increment can be relatively large because of conditional stability of the implicit time integrator and static solutions can be obtained by natural characteristics of the method. To solve the governing equations by applying the unbalanced forces and computing the corresponding displacements, the Newton–Raphson method was used. The advantage of this incrementation technique is its accuracy and rapid (but conditional) convergence.

## 5. Results and Discussion

**Figure 8:**
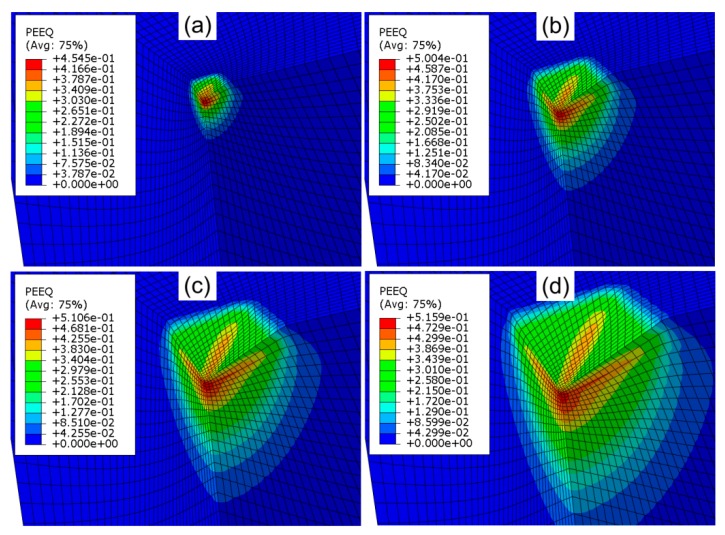
Distribution of equivalent plastic strain in the impression area at frictionless conditions and for indentation depths: (**a**) 0.025 mm, (**b**) 0.05 mm, (**c**) 0.075 mm and (**d**) 0.1 mm (identical scales).

When the load was increased to a critical value, the onset of plasticity occurred beneath the surface of material deformed. The maximum value of equivalent plastic strain was located at a certain depth value under the impression surface (Figure 8). The increase in the friction coefficient value increased the deepness of the plastically deformed region. However, the difference in the values of the equivalent plastic strains for the analyzed range of the friction coefficient were smaller than those observed by Antunes et al. [11] who studied the indentation of Vickers pyramid at an indentation load of 10–20 mN and friction coefficients of μ = 0.04 and μ = 0.24.

**Figure 9:**
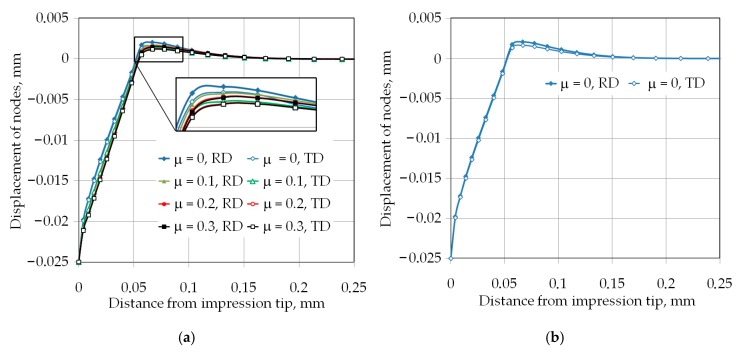
(**a**) Displacement of the nodes along the rolling direction (RD) and (TD) for indentation depth 0.025 mm and (**b**) effect of the material anisotropy on the pile-up effect.

**Figure 10:**
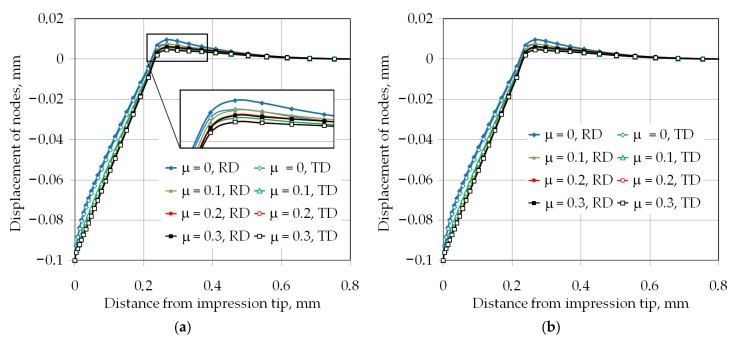
(**a**) Displacement of the nodes along RD and TD for indentation depth 0.1 mm, and (**b**) effect of the material anisotropy on the pile-up effect.

For analyzed frictional conditions and indentation depths, the pile-up effect was observed. Figure 9 and Figure 10 show the displacement of the nodes laying along the rolling direction (RD) and transverse to the rolling direction (TD) in the z-direction (Figure 6), under loading conditions. Increasing the friction coefficient led to decreased pile-up values. It can be associated with the difficulty of the movement of the workpiece material from the face of the indenter towards to the edge of impression. The increase in pile-up height with indentation depth would introduce an indentation size effect if the hardness was corrected for the pile-up effect. The method to obtain the hardness value corrected for pile-up from the bulk behavior was proposed by Gale and Achuthan [39]. Both pile-ups in the RD and TD are not symmetrical around the indenter symmetric edges. Furthermore, the width of the pile-ups differed from each other at the two perpendicular directions of measurement. In all analyzed cases of the friction conditions and indentation depths, the height of pile-up was greater in the RD than in the TD. This might be directly associated with material anisotropy if the plastic and elastic properties were different based on the directions.

Exemplary distributions of the pile-up heights have been presented in Figure 9b and Figure 10b for two extreme conditions of simulations, i.e., μ = 0, *h* = 0.025 mm; and μ = 0, *h* = 0.1 mm respectively. It is clear from these two figures, which were at frictionless conditions, that the main phenomenon that caused the difference in the pile-ups height and width was the material anisotropy. In such conditions, the fractional area of contact around the pyramid indenter alters the hardness produced in bulk deformation.

**Figure 11:**
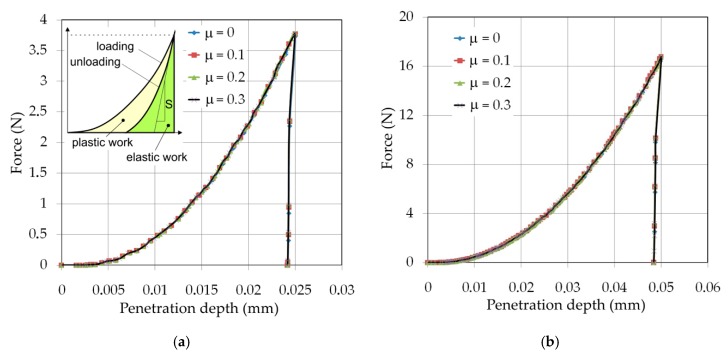

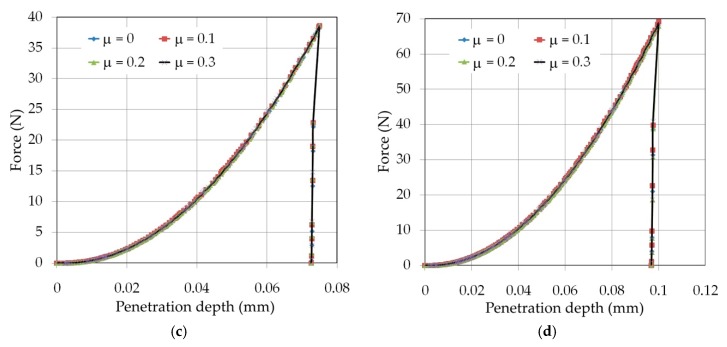
Load–displacement characteristics for penetration depths: (**a**) 0.025 mm, (**b**) 0.05 mm, (**c**) 0.075 mm and (**d**) 0.1 mm.

The load–displacement curves of Vickers hardness indentation are shown in Figure 11, where the hysteresis curve in Figure 11a is a typical curve for elastic-plastic materials. The plastic deformation is the main phenomenon observed during indentation. In the case of all indentation depths and friction conditions, the elastic response of the material is described by the very steep line. The effect of the frictional conditions on the force is negligible.

The values of the maximum force in the case of indentation depth of h = 0.025 mm are equal to 3.732 N, 3.773 N, 3.778 N and 3.785 N for the friction conditions of *μ* = 0, *μ* = 0.1, *μ* = 0.2 and *μ* = 0.3, respectively, so, the percentage difference in load force did not exceed 1.42%. Increasing he indentation depth to *h* = 0.1 mm caused a decrease of the percentage difference in maximum load force for friction conditions analyzed to the value of 0.94%. The unloading stiffness of the sheet material as the initial slope of the unloading curve after unloading is very similar for all samples because the plastic deformation of the sheets does not change the elastic properties of the material [40,41].

**Figure 12:**
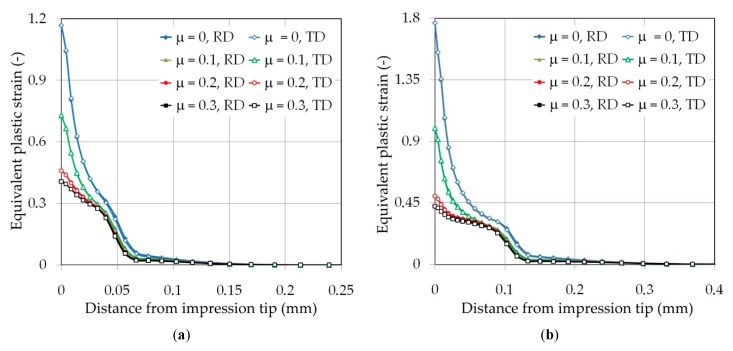

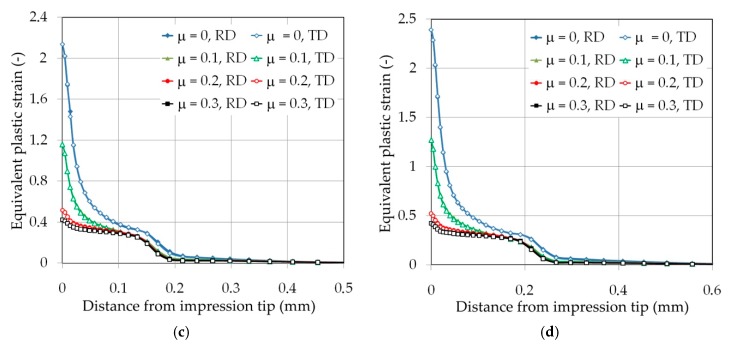
Effect of the material anisotropy on the distribution of the equivalent plastic strain along the rolling direction (RD) and transverse to the rolling direction (TR) of the sheet metal at indentation depths of (**a**) 0.025 mm, (**b**) 0.05 mm, (**c**) 0.075 mm and (**d**) 0.1 mm.

Figure 12 shows the distribution of the equivalent plastic strain in the area of impression along the RD and TD under loading. A clear effect of the friction conditions on the value of equivalent plastic strain can be revealed, especially at the tip of the impression. An increase in the coefficient of friction causes a limitation of the material flow from the tip of the indenter into the impression edge zone. As a consequence, the distribution of equivalent plastic strains predicted at friction was characterized by a more flat shape. The effect of the friction coefficient value on the value of equivalent plastic strain in the area of impression tip was distinct. The difference between the values of equivalent plastic strain measured at RD and TD increased with increasing distance from the impression tip and reached 8%–19% depending on friction conditions and indenter displacement.

**Figure 13:**
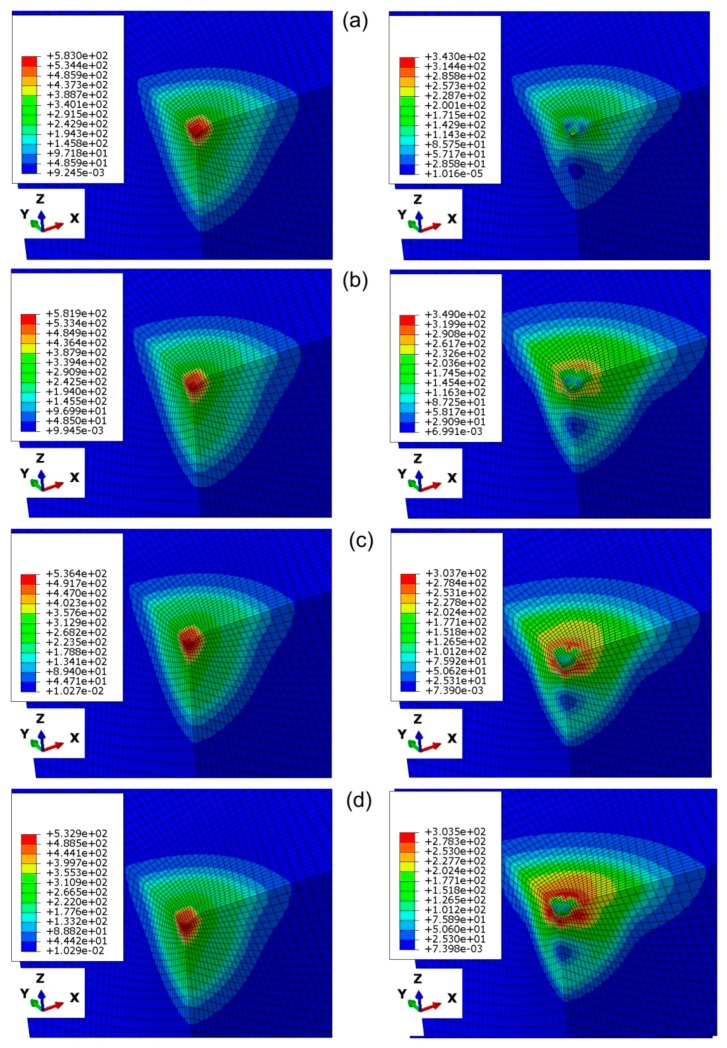
Distribution of equivalent stress (in MPa) under maximum displacement (left-hand side) and after unloading (right-hand side) state for the indentation depth of 0.025 mm and friction coefficients: (**a**) 0, (**b**) 0.1, (**c**) 0.2 and (**d**) 0.3.

**Figure 14:**
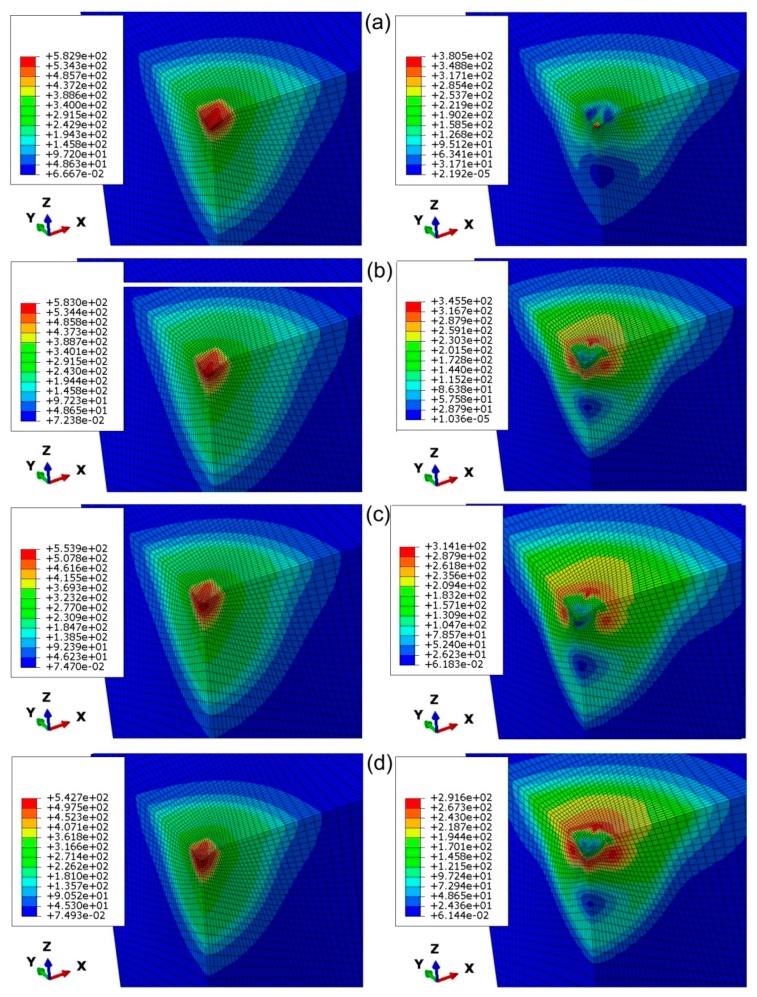
Distribution of equivalent stress (in MPa) under maximum displacement (left-hand side) and after unloading (right-hand side) state for the indentation depth of 0.05 mm and friction coefficients: (**a**) 0, (**b**) 0.1, (**c**) 0.2 and (**d**) 0.3.

The distribution of equivalent plastic stress under the indenter is quite dependent on the value of the friction coefficient (Figure 13 left). This is in accordance with the observations of Antunes et al. [11]. A similar conclusion might be found after analysis of the distributions of equivalent plastic stress under the maximum penetration depth of the indenter (Figure 14 left). Increasing the friction coefficient value led to reduction of the maximum equivalent plastic stress (Figure 13, Figure 14 left). It is obvious that the maximum value of equivalent plastic stress under maximum displacement was observed in the area of highest depth of the impression.

While unloading the impression, a part of the deformation zone remained plastic and the rest of the deformation recovered. The elastic recovery of the stress distribution causes the springback of the material and change in the diagonal dimensions of the imperfection, in the case of anisotropic materials [37]. For low values of the friction coefficient, the maximum value of the equivalent plastic stress under unloading was the highest (Figure 13, Figure 14 right). In the case of analyses that had taken into account the frictional conditions, the region of maximum residual stresses was located in the subsurface (Figure 13b–d right; Figure 14b–d right).

## 6. Conclusions

The hardness testing is the easiest and the fastest method to characterize the elastic-plastic properties of the metallic materials. The aim of this paper was to conduct a numerical investigation of the effect of contact conditions on the response of material under indentation of Vickers pyramid into anisotropic material. The study of the friction conditions presented has shown the importance of its consideration in the analysis of pile-up effect and consequently in the contact area evaluation. The following conclusions are drawn from the research:

- The hardness values seem to be influenced by the value of friction coefficient due to the pile-up phenomenon observed;
- The increasing of the friction coefficient led to decrease of pile-up value. Moreover, the width of the pile-ups differed from each other at the two perpendicular directions of measurement. This phenomenon may be attributed primarily to the anisotropy of material properties, and in a lesser extent with the friction coefficient value;
- Frictional conditions did not show a significant effect on the maximum force and the character of load-displacement curves;
- Although the effect of the friction coefficient value on the value of equivalent plastic strain in the area of impression tip is distinct, the difference between the values of an equivalent plastic strain measured at RD and TD increased with increasing distance from the impression tip and reached 8%–19% depending on friction conditions and indenter displacement;
- Increasing the friction coefficient value led to reduction of the maximum equivalent plastic stress observed under maximum load. While unloading, the elastic recovery of the stress distribution caused the springback of the material and anisotropically changed the diagonal dimensions of the imperfection. Frictional regime between indenter and workpiece caused that the region of maximum residual stresses to be located in the subsurface.

## Figures and Tables

**Table 1:** The chemical composition of DC04 steel sheet (wt.%).

C	Mn	Si	P	S	Al	Cu	Ti	Nb	Fe
0.04	0.28	0.012	0.008	0.006	0.017	0.012	0.0006	0.0029	balance

**Table 2:** Mechanical properties of the DC04 steel sheet.

Orientation	*R_p0.2_*, MPa	*R_m_*, MPa	*K*, MPa	*n*	*r*
0°	182.1	322.5	549.3	0.214	1.751
45°	196	336.2	564.9	0.205	1.124
90°	190	320.9	541.6	0.209	1.846
Average value	191.2	328.95	555.17	0.208	1.461

**Table 3:** The maximum indentation force for the analyzed finite element (FE)-based models.

	Number of Elements			
	46202	79507	124384	238140
Maximum indentation force, N	16.7837	16.7328	16.6964	16.6959
